# Seasonally Dependent Change of the Number of Fractures after 50 Years of Age in Poland—Analysis of Combined Health Care and Climate Datasets

**DOI:** 10.3390/ijerph19159467

**Published:** 2022-08-02

**Authors:** Kinga Jedynasty, Mariusz Zięba, Jakub Adamski, Marcin Czech, Piotr Głuszko, Dariusz Gozdowski, Agnieszka Szypowska, Andrzej Śliwczyński, Magdalena Walicka, Edward Franek

**Affiliations:** 1 Department of Internal Diseases, Endocrinology and Diabetology, Central Clinical Hospital MSWiA, 02-507 Warsaw, Poland; kingaeleven@hotmail.com (K.J.); m_walicka@wp.pl (M.W.); 2 Ministry of Health, 00-952 Warsaw, Poland; mariusz.zieba3@gmail.com (M.Z.); adamski.jakub@gmail.com (J.A.); 3 Department of Pharmacoeconomics, Institute of Mother and Child, 01-211 Warsaw, Poland; marcin.czech@biznes.edu.pl; 4 Department of Rheumatology, National Institute of Geriatrics, Rheumatology, and Rehabilitation, 02-637 Warsaw, Poland; zruj@mp.pl; 5 Department of Biometry, Warsaw University of Life Science, 02-787 Warsaw, Poland; dariusz.gozdowski@sggw.pl; 6 Department of Pediatrics, The First Faculty of Medicine, Medical University of Warsaw, 02-091 Warsaw, Poland; agnieszka.szypowska@gmail.com; 7 Satellite Campus in Warsaw, University of Humanities and Economics in Lodz, 01-943 Warsaw, Poland; andrzej.sliwczynski@cskmswia.pl; 8 Department of Human Epigenetics, Mossakowski Medical Research Institute, 02-106 Warsaw, Poland

**Keywords:** fracture, epidemiology, temperature, climate, season, weather

## Abstract

Aim: The incidence of fractures correlates with many independent and interrelated factors. The aim of the study was to examine trends in fracture incidence and to find possible reasons for changes. Materials and methods: A complete dataset of Polish population aged above 50 from the National Heath Fund—which is a single, state-owned payer for the health service procedures in Poland—covering the years between 2010 and 2015 was analyzed along with climate dataset. Results: The analysis indicated that there was a substantial and statistically significant decrease in the incidence of forearm and hip fractures (*p* = 0.007 and 0.007, respectively). On the other side, there was a statistically significant increase in incidence of humerus and lumbar fractures (*p* = 0.002, *p* < 0.001, respectively). The observed changes (especially decrease in forearm and hip fracture incidence) happened mostly in the cold season and were correlated to mean-temperature changes during the assessed time period. Conclusion: In the analysis based on the dataset obtained from fracture-related database collected in Poland in the years 2010–2015 in the population of patients over 50 years of age, we observed that the changes of fracture incidence during the observation period are associated with and may be dependent on the season (warmer versus colder) and on mean temperature increase during the observation period.

## 1. Introduction

The incidence of fractures is very high. It was assessed that, in 2019, incidence of all fractures totaled 178 million globally [1]. The absolute incidence has increased globally by 33.4% from 1990 [1] as a consequence of increasing number and percentage of elderly subject in the population. The absolute number of people older than 65 years increased from 327.6 million in 1990 to 673.7 million in 2017 (6.1–8.8% of the whole population) and this number is predicted to be twice as high in 2050 [2]. Increased mobility of elderly and increased trauma rate [3] may also play a role—it is predicted that over the coming decades injuries will be a major cause of mortality and disability [4,5].

Incidence of fractures worldwide follows a bimodal pattern. It is high in youth [6], then lower and rather stable in adults, and increases again sharply after menopause in women and in elderly people of both sexes [7]. Whereas, among youths, fractures are mostly result of a violent injuries (such as vehicle collisions or falling from considerable height), fractures in the elderly are mainly low-energy (or fragility) fractures and are usually defined as fractures resulting from falling from standing height. The reason of that is that healthy bones in young subjects are resilient and can withstand surprisingly powerful impacts. With age, however, bone mass and strength decrease and osteoporosis may develop. It is characterized by reduced bone mass and disruption of bone architecture, resulting in increased bone fragility and increased fracture risk [8]. Based on WHO diagnostic criteria (T-score less than or equal to −2.5 SD), in 2010 approximately 22 million women and 5.5 million men aged between 50 and 84 years of age in the EU had osteoporosis [9].

The most common fractures associated with osteoporosis are those of the spine, forearm, hip, and humerus; however, other fractures (such as wrist fractures) after the age of 50 years are associated with low BMD and may be regarded as osteoporotic [10]. The incidence of fragility fractures increases markedly with age, although the rate of rise with age (and, therefore, the proportion of fractures at any site) differs for different fractures [11,12]. For example, hip fractures are rare at the age of 50 years, but become the predominant osteoporosis fracture in the eighth decade of life. Comparatively, incidence of distal forearm fractures peaks relatively early (around 65 year of age) and after that remains stable [9].

Several studies evaluated the effect of weather conditions on the incidence of fractures across the countries and regions [13,14,15,16,17,18,19,20,21,22,23,24,25]. For example, a systematic review summarizing the association between climate and hip fractures indicated that hip fracture incidence seems to correlate with seasonal changes, with the greater incidence of hip fractures occurring in winter. Temperature seems to be the variable that best represents these seasonal changes [14]. However, to our knowledge no evidence has been published regarding fracture incidence, its relationship with seasonal weather changes in Poland, and regarding a possible impact of the mean temperature increase that has occurred in the last years on the fracture incidence.

The aim of our study was an attempt to assess the presence of and reasons for changes of seasonal variations of fracture incidence in different locations (humerus, wrist, forearm, femur, hip, lumbar- and thoracic spine) in the years 2010–2015 in Poland.

## 2. Materials and Methods

The study was a part of the project entitled: Maps of Health Needs: A Systemic and Implementation Analyses Base, co-financed by the European Union from the European Social Fund as part of the Operational Program Knowledge Education Development. The project was carried out and published by the Analyses and Strategies Department of the Ministry of Health in Poland [26].

Data regarding incident fractures were obtained from the National Health Fund (NHF) database. NHF is a public payer financing medical procedures in Poland. Almost all Polish citizens are insured in the NHF that maintains electronic register of services that were contracted with and reported to it. The data transferred to the NHF include among others the patient’s name, sex, age, unique personal ID number (PESEL), diagnosis according to ICD-10, and ID of the diagnosis-related group (DRG) [27]. As transfer of the data is required for payment, practically all incident fractures that are diagnosed are also reported to the NHF.

However, as the information whether the fracture was a low-energy fracture is not reported and not available in the NHF database, it was decided that only patients aged 50 and above (among whom the probability that the fracture was an osteoporotic one is very high [28]), would be included in the analysis. For the purpose of this study, only first fractures in the given location that happened in the examined time period (and were reported to the NHF from the ambulatory and stationary health care units) were taken into account. The reason for that is that ambulatory visits after a fracture even within a long period of time (e.g., for control of healing), are reported with the same codes. Reporting them as incident fractures would have falsely increased the number of fractures even 3–4 times. The fractures were identified according to ICD-10 classification: humerus (ICD10 codes: S42.2, S42.3, S42.4), femur (S72, S72.0, S72.1, S72.2), lumbar (S32.0, S32.7) and thoracic vertebra (S22.0, S22.1), wrist (S62, S62.0, S62.1, S62.2, S62.3, S62.4, S62.5, S62.6, S62.7, S62.8) and forearm (S52, S52., S52.0, S52.1, S52.2, S52.3, S52.4, S52.5, S52.6, S52.7, S52.8, S52.9). Patients diagnosed at any time with malignant neoplasm (any of ICD-10 diagnosis with a code beginning with C) were excluded.

Based on the differences of fractures incidence observed in the examined years the months of the year have been grouped in two periods. One of them comprised November to March (‘winter’) and the other April to October (‘summer’).

Meteorological data have been gathered from the NOAA (National Oceanic and Atmospheric Administration) database [29]—this easily accessible database collects information from official sources around all the world. For Poland it gathers them from the IMGW—i.e., the Institute of Meteorology and Water Management. It is also possible to gather the data directly from the IMGW website, but the data management is cumbersome. Once a check has been made on several data points between the two databases and the identity has been verified, the NOAA database was utilized, thus providing all data in tabular excel or csv formatting. As there were no geolocalizations available for the health data, after evaluating several options, the meteorological station of Warsaw-Okęcie has been selected as a proxy for the whole country. One of the main drivers for this choice, apart from its centrality in the country, was the fact that the daily values from this meteorological station were accessible for the whole study period without gaps.

### Statistical Analysis

The analyses were conducted separately for winter (November–March) and summer (April–October) seasons. Trend analysis of number of various types of fractures in years 2010–2015 was evaluated using Pearson’s correlation and linear regression. Moreover, joinpoint regression [30] was applied to evaluate annual percent change (APC) of number of fractures together with confidence intervals (CI 95%). Because of annual variability present in the colder (November–March) but not warmer (April–October) seasons, the analyses were conducted separately for those months (seasons). Multiple linear regression was applied for evaluation of relationships between year, season, and gender with mean daily number of incident fractures. Relative risk together with confidence intervals was calculated to evaluate differences between the colder and the warmer seasons. On the basis of coefficients of regression mean annual and seasonal changes (increases or decreases) were estimated. A long term trend in the number of incident fractures was evaluated using time series based on additive model where moving average and seasonal indices (values are differences between number of incident fractures for individual month and moving average for one year).

Additionally, the relationships between selected weather variables—i.e., mean annual and monthly temperatures or number of days with snow—and number of fractures were analyzed in the period of 2010–2015. Analysis of linear regression was applied to evaluate relationships between weather variables and daily number of fractures (daily fractures that happened in those particular periods were treated as dependent variable). Significance level for all statistical analyses was set at 0.05 probability level. The analyses were performed in Statistica 13 software and Joinpoint Trend Analysis Software (version 4.9.1.0) developed by US National Cancer Institute.

## 3. Results

**Figure 1:**
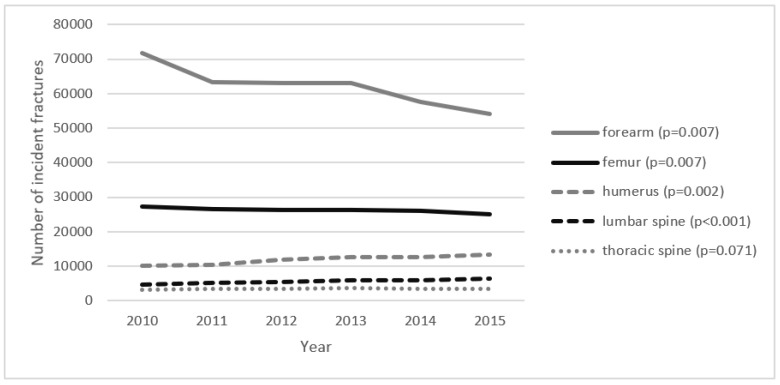
Absolute number of incident fractures in the years 2010–2015. Significance level assessed in the linear regression analysis is given in the brackets.

In the period between 2010 and 2015, there were 373,139 forearm fractures, 171,701 humerus fractures, 157,443 hip fractures, 33,778 lumbar vertebral, and 20,674 thoracic vertebral fractures. The change of the number of above listed fractures in the particular years is shown in the Figure 1, and the results of a joinpoint regression analysis are shown in Table 1.

As it can be seen, there was a substantial decrease in the incidence of forearm and hip fractures, that was statistically significant. On the other side a statistically significant increase in incidence of humerus and lumbar fractures was observed. The incidence of thoracic spine fractures was not statistically different in the examined years. The number of particular fractures stratified by sex and year is given in Table 2.

**Figure 2:**
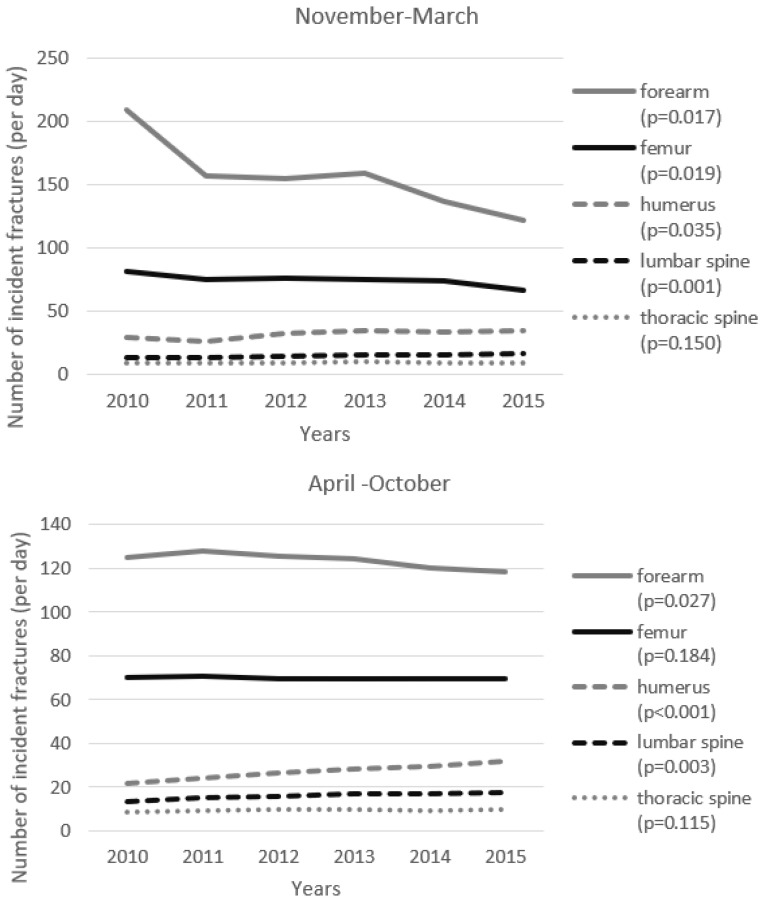
Number of incident fractures (per day) in time periods between November and March and between April and October. Significance level assessed in the linear regression analysis is given in the brackets.

**Figure 3:**
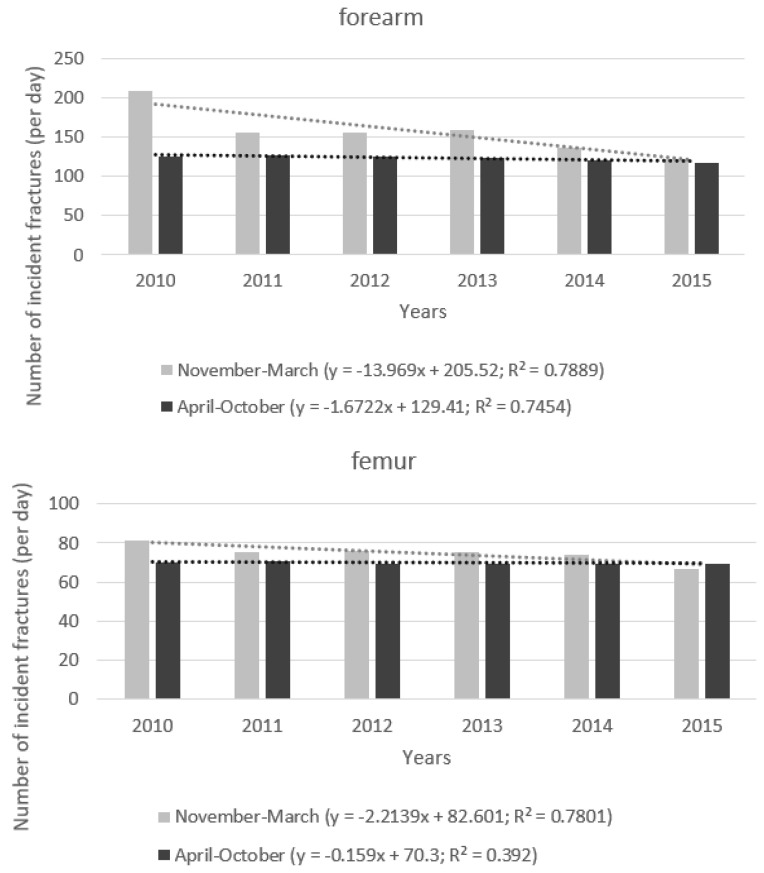
Trends of changes of forearm and hip fractures’ incidence (per day) assessed separately for the months November–March and April–October. *x*-axis—year of the study; *y*-axis—daily number of incident fractures. Difference between the slopes is statistically significant (*p* = 0.013 for forearm, and 0.009 for hip fractures, respectively).

The changes of fracture incidence during the observation period depended on the season. For example, the incidence of forearm fractures in the subsequent years decreased substantially between November and March, whereas the decrease between April and October was much less prominent (Figure 2). The different slopes of decrease in the fracture incidence are shown in the Figure 3. Both trends were significant (*p* = 0.017 for November–March and *p* = 0.027 for April–October), however the mean decrease in the number of fractures in the colder season was equal to almost 14 fractures per day, whereas in the warmer season it was only 1.7 fracture per day. Statistical comparison of slopes has proven a significant difference (*p* = 0.013) between these two regression functions (Figure 3). Similar pattern is visible for hip fractures. Number of fractures decreased for both seasons, significantly for the colder (*p* = 0.019) and non-significantly (*p* = 0.184) for the warmer one. In the colder season, mean decrease per year was 2.2 fractures per day, whereas in the warmer season the decrease was much lower (0.16 fracture per day). This difference was statistically significant (*p* = 0.009, Figure 3). Similar results were achieved when relative risk of fracture during the colder season compared to warmer season (Table 3). As can be seen, the risk of fracture during winter was significantly increased in case of forearm, femur, and humerus fractures. Risk of lumbar spine fracture was comparatively significantly lower and risk of thoracic spine fracture similar. These results remained significant after adjustment for sex (not shown).

**Figure 4:**
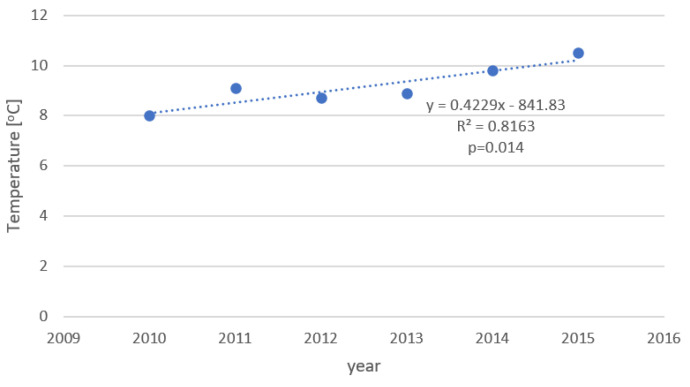
Changes of mean annual temperature between 2010 and 2015. *x*-axis—year of the study, *y*-axis—mean annual temperature.

**Figure 5:**
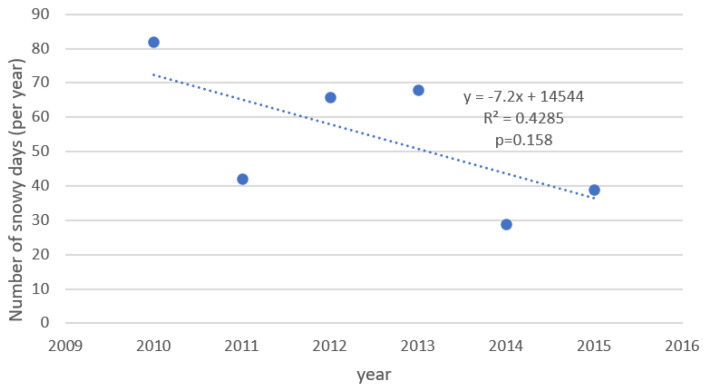
Changes of the number of snowy days in the given year between 2010 and 2015. *x*-axis—year of the study; *y*-axis—yearly number of snowy days.

The changing number of fractures was also related to the weather parameters. Figure 4 and Figure 5 show the changes of mean annual temperature and of the number of snowy days in the observation period. Table 4 shows a regression analysis of different weather variables and the number of particular fractures (for period November–March). As it can be seen, the mean annual temperature was significantly negatively correlated with the number of hip and forearm fractures (the lower temperature the higher number of fractures). Similar correlations were found between hip and forearm fractures and mean temperature in January and in December. There was also a positive, significant correlation between the number of forearm fractures and the number of days with snow (per year). Besides, a positive significant correlation between mean annual temperatures with number of lumbar spine fracture was observed, a similar trend for the thoracic spine was not significant. The results and the trends were similar for men and women.

## 4. Discussion

In this analysis based on the assessment of the dataset obtained from fracture-related database collected in Poland in the years 2010–2015 in the population of patients over 50 years of age and the dataset comprising the meteorological data, we observed that the changes of fractures incidence during the observation period are associated with—and may be dependent on—the season (warmer versus colder), especially that the substantial decrease in forearm fracture incidence happened above all in the winter.

Several studies evaluated seasonality and the effect of weather conditions on the incidence of fractures across the countries and regions [17,18,19,20,21,22,23,24,25,31,32,33,34,35]. Practically all studies show an increased incidence of fractures in the elderly in the colder season, although monthly distribution may be different worldwide. In the northern hemisphere, more fractures happen in December, January, and February. From the other side, in the southern hemisphere, more fractures occur in June, July, and August, even though these temperatures in winter months are higher in comparison with the northern hemisphere [35]. Many studies also show a direct relationship between fracture incidence and temperature, not only with season [22,32].

The reason for that is probably the increased incidence of falls in the cold season, related to more frequent occurrence of snow and ice cover, or even water in the streets, which increase the probability of slips and falls [36]. Falls are frequently the direct cause of fracture. Falls are common: one-third of people over the age of 65 fall annually, with approximately 10–15% of falls in the elderly resulting in fracture [9]. Colles’ fracture is the type of peripheral fracture that is most frequently associated with falls [37]. Other extra-vertebral (humeral, wrist, pelvis, and hip) fractures also frequently result from the combined effects of osteoporosis and the fall [38,39,40,41,42,43], whereas vertebral fractures are less fall-dependent [44,45].

However, whereas the relationship between a cold (winter) season and increased incidence of fractures has been explored quite thoroughly, there are no published observations describing changing trends in fractures incidence in specific years and relating them to the changing weather.

We have observed that the incidence of forearm fractures decreased substantially between November and March (*p* = 0.017). The decrease between April and October was also significant (*p* = 0.027), but much less prominent (Figure 2 and Figure 3) and a statistical comparison of slopes for these two regression functions has proven a significant difference (*p* = 0.013).

A similar pattern is visible for hip fractures. The number of fractures decreased for both periods, significantly in the colder (*p* = 0.019) and non-significantly (*p* = 0.184) in the warmer season. The decrease in a daily number of fractures in the winter was almost 14 times greater than in the warmer season. This difference was statistically significant (*p* = 0.009). Differences of the changes of fracture incidence between warmer and colder season for humerus (*p* = 0.396) and spine fractures (*p* = 0.840 and 0.849 for lumbar and thoracic vertebra, respectively) were not significant.

As mentioned before, incidence of fractures increases with lower temperatures. Indeed, temperature seems to be the simple indicator of seasonal changes. Having the information on both the incidence of fractures over the longer time period and the mean annual temperature may enable obtaining new insights on possible correlation between fractures and the seasonal temperature changes. According to our knowledge, there was no research until now looking at seasonal changes of the fracture incidence and their changes over years.

As we expected, those changes were indeed interrelated. As can be seen in Table 1, temperature in winter months, mean annual temperature, and number of snowy days correlated significantly with the incidence of some fractures. The obvious link between weather parameters and fracture changes are falls, that occur less frequently when there is no risk of slide. This is in concordance with our observation of a decrease in incidence of fractures which depend on falls—i.e., forearm and hip fractures. The higher the temperature, the lower the number of days with snow fall, leading to a lower number of those fractures.

Interestingly, a positive significant correlation between mean annual temperatures with number of lumbar spine fracture was observed, a similar trend for thoracic spine was not significant. This observation was unexpected, and the explanation for that might not be easy. The relationship is probably not connected with falls, as the current status of research in this field shows that vertebral compression fractures occur frequently in the absence of a fall [43], besides the relationship would be rather negative. One possible explanation is that the more warmer days there are in the winter, the more people may work outside their homes lifting heavy objects that may exert an excessive force on their lumbar, but not necessarily on their thoracic spine. This hypothesis however would have to be confirmed.

This study has many limitations. First, the observation period is relatively short, although it seems that even in such a short period the temperature increased and the number of snowy days decreased, and even in such a short period the observed temperature changes were significant. Another limitation is that the temperature and number of snowy days may differ in particular regions of Poland, and that the incidence of fractures in the particular regions may change in a different way. For example, in southern Poland more days during the winter season are snowy and the temperature is lower than in the northern part of the country. As the data from meteorogical stations are limited to some parts of Poland only, even using the temperature and snow measurements from all of them it would be impossible to provide the exact relationships for smaller areas, we decided to accept this limitation.

Another limitation is that only about one-third (or even less) of vertebral fractures come to clinical attention. The reason for this issue is that, in contrast to other bone fractures, they are usually low symptomatic or asymptomatic. Therefore, in the retrospective analysis of the vertebral fracture rates based on ICD-10 classification, one should take into account that ICD and DRG codes for vertebral fractures may be used when an incident fracture occurs as well as if the prevalent fracture is detected during, for example, back pain diagnosis. Therefore, the numbers shown in this study are not real incidence but rather a mixture of incidence and prevalence.

Finally, there is no information on the reasons and causes of a fracture in the NHF database, as well as no data regarding may risk factors, such as weight or low bone mineral density. There are also no data that would allow us to distinguish low and high energy fractures. We have excluded all patients with a diagnosis of a malignant neoplasm, which may be connected with fractures, and probably most of the fractures in the examined population were osteoporotic, as it is assumed that fractures in subjects after 50 years of age in typical localizations are usually related to low BMD [10], but the real proportion of low- and high energy fractures is unfortunately unknown.

From the other side, the strength of the study is that in Poland—where the health service costs connected with fractures are covered by National Health Fund as one single payer—all major fractures are registered in the NHF database. The study showing the high incidence of fractures also shows the importance of fracture prevention in Poland. Every additional decrease of even 10% would decrease the absolute number of fractures by many thousand, decreasing the costs, improving quality of life and mortality in the elderly. Our results also trigger new perspectives for analyses and interpretation of trends of epidemiology of fractures and their changes with regard to anticipated long-term climate changes (climate warming), which might become an important factor influencing the numbers of fractures in elderly, next to other issues such as: aging population, osteoporosis, progress of medical treatments, and implementation of preventive programs. Taking into account the health-related and economic burden connected with fractures, this area of further research and observation will definitely continuously gain on importance.

## 5. Conclusions

In the analysis based on the dataset obtained from fracture-related database collected in Poland in the years 2010–2015 in the population of patients over 50 years of age, we observed that the changes of fractures’ incidence during the observation period are associated with and may be dependent on the temperature and on the season (warmer versus colder), especially that the substantial decrease in forearm fracture incidence occurred almost exclusively in the winter.

## Figures and Tables

**Table 1:** Joinpoint regression analysis assessing the significance of the change of number of the particular fractures in the years 2010–2015. APC—annual percent change. CI—confidence interval. *p*—level of significance.

	Segment	Lower Endpoint	Upper Endpoint	APC	Lower CI	Upper CI	Test Statistic (t)	*p*
Humerus	1	2010	2015	6.0	3.6	8.5	7.0	0.002
Femur	1	2010	2015	−1.4	−2.2	−0.6	−5.1	0.007
Lumbar spine	1	2010	2015	5.5	4.1	6.9	10.9	<0.001
Thoracic spine	1	2010	2015	1.7	−0.2	3.6	2.5	0.07
Forearm	1	2010	2015	−4.7	−7.1	−2.3	−5.4	0.006

**Table 2:** Absolute number of particular fractures stratified by sex and year.

	Humerus	Femur	Lumbar Spine	Thoracic Spine	Forearm
	Women				
2010	7198	19,415	2849	1751	57,011
2011	7401	18,650	3100	1819	49,487
2012	8429	18,621	3282	1981	49,494
2013	8981	18,574	3624	2026	50,030
2014	9087	18,349	3747	2013	45,478
2015	9640	17,578	3848	2063	42,387
2010–2015	50,736	111,187	20,450	11,653	293,887
	Men				
2010	2911	7876	1941	1472	14,896
2011	2934	7851	2122	1520	13,739
2012	3400	7749	2266	1548	13,546
2013	3546	7669	2254	1554	13,099
2014	3500	7687	2295	1467	12,190
2015	3701	7424	2450	1460	11,782
2010–2015	19,992	46,256	13,328	9021	79,252
	Total population				
2010–2015	70,728	157,443	33,778	20,674	373,139

**Table 3:** Relative risk of particular fractures during exposure to cold season versus warm season. CI—confidence interval. *p*—level of significance.

	Humerus	Femur	Lumbar Spine	Thoracic Spine	Forearm
Relative risk	1.170	1.073	0.910	0.965	1.267
95% CI	1.111–1.231	1.039–1.109	0.847–0.976	0.881–1.056	1.238–1.297
*p*	<0.001	<0.001	0.009	0.433	<0.001

**Table 4:** Regression equations (*p*-values in brackets) between weather variables and daily number of fractures.

	Humerus	Femur	Lumbar Spine	Thoracic Spine	Forearm
Mean temperaturę in January	36.42 + 0.55 × (0.518)	78.97 − 0.92 × (0.222)	15.04 + 0.15 × (0.679)	10.07 + 0.10 × (0.212)	184.82 − 5.73 × (0.329)
Mean temperature in February	32.91 + 0.60 × (0.539)	74.58 − 0.83 × (0.112)	15.17 + 0.55 × (0.023)	9.61 + 0.17 × (0.330)	174.55 − 4.28 × (0.467)
Mean temperature in March	31.18−0.68 × (0.350)	73.70 − 0.87 × (0.177)	13.63 + 0.15 × (0.619)	8.78 + 0.01 × (0.902)	164.70 − 9.19 × (0.030)
Mean temperature in November	24.40 + 0.50 × (0.736)	64.95 + 0.68 × (0.310)	15.17 − 0.10 × (0.881)	8.08 + 0.20 × (0.453)	95.31 + 1.94 × (0.704)
Mean temperature in December	36.00 − 0.56 × (0.170)	79.50 − 3.25 × (0.032)	14.94 + 0.26 × (0.137)	9.20 − 0.07 × (0.453)	174.72 − 16.48 × (0.002)
Mean temperature for the cold season (November–March)	30.62 + 1.05 × (0.268)	77.40 − 2.52 × (0.005)	13.86 + 0.71 × (0.044)	9.18 + 0.07 × (0.556)	172.00 − 15.22 × (0.014)
Number of snowy days (per year)	33.26 − 0.03 × (0.739)	66.21 + 0.16 × (0.123)	16.72 − 0.04 × (0.264)	9.38 + 0.00 × (0.805)	92.66 + 1.18 × (0.044)
Mean annual temperature	0.65 + 3.09 × (0.070)	91.85 − 2.18 × (0.005)	1.52 + 1.52 × (0.023)	7.29 + 0.23 × (0.258)	273.54 − 14.87 × (0.003)

## Data Availability

The data will be available upon reasonable requests form Dariusz Gozdowski (dariusz.gozdowski@sggw.pl).
